# Supplementary material for: Cell Lysis Directed by SulA in Response to DNA Damage in Escherichia coli
Source: Int J Mol Sci. 2021 Apr 26;22(9):4535. doi: 10.3390/ijms22094535 (PMC8123628; doi:10.3390/ijms22094535)
Supplement: Supplementary file 1 [file ijms-22-04535-s001.zip › ijms-1163905-supplementary.pdf]

Supplementary Materials

Cell lysis directed by SulA in response to DNA damage in *Escherichia coli*

Masayuki Murata[1], Keiko Nakamura[2], Tomoyuki Kosaka[1,3], Natsuko Ota[1], Ayumi Osawa[1], Ryunosuke Muro[2], Kazunari Fujiyama[2], Taku Oshima[4], Hirotada Mori[4], Barry L. Wanner[5] and Mamoru Yamada[1-3]*

[1]Life Science, Graduate School of Science and Technology for Innovation, Yamaguchi University, Ube 755-8611, Japan
[2]Applied Molecular Bioscience, Graduate School of Medicine, Yamaguchi University, Ube 755-8505, Japan
[3]Research Center for Thermotolerant Microbial Resources, Yamaguchi University, Yamaguchi 753-8315, Japan
[4]Department of Biotechnology, Toyama Prefectural University, 5180, Kurokawa, Imizu, Toyama 939-0398, Japan
[5]Graduate School of Information Science, Nara Institute of Science and Technology, 8916-5 Takayama, Ikoma, Nara 630-0192, Japan
[5]Department of Microbiology, Harvard Medical School, Boston, MA 02115, USA

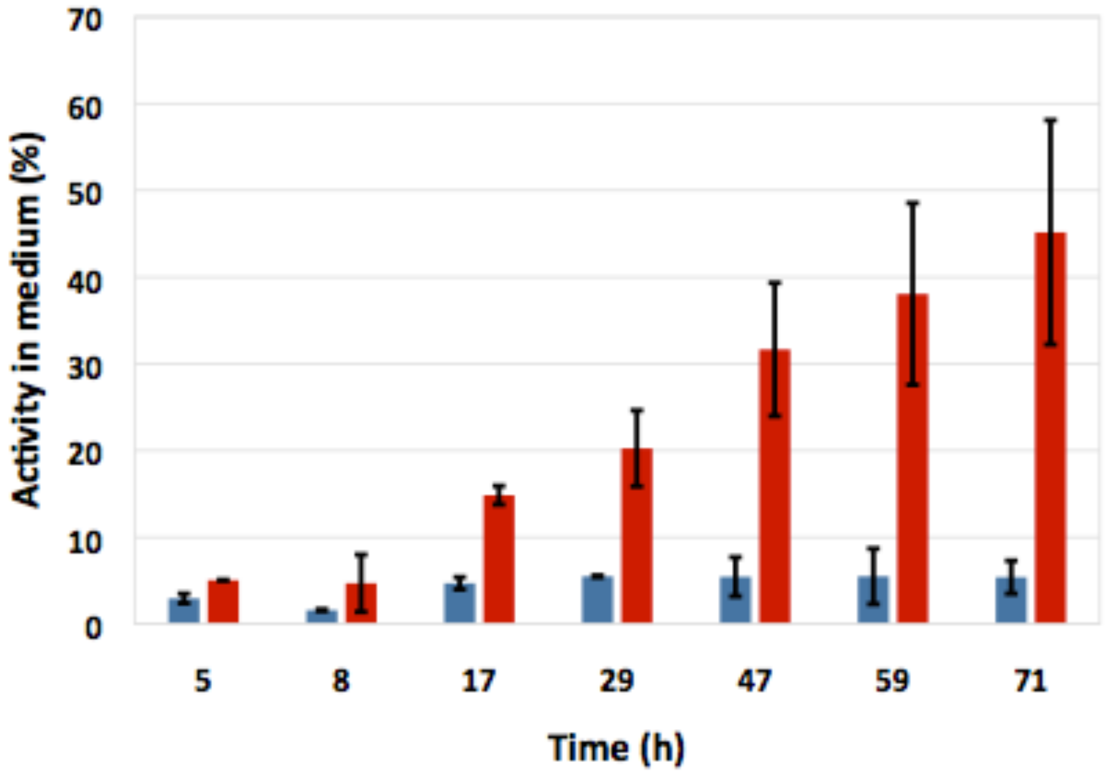

Figure S1. β-Galactosidase activity in the medium fraction.
W3110N *lon*::*kan* cells harboring pBAD24 or pBAD-*sulA* were grown in LB medium containing ampicillin and kanamycin. L-arabinose was added at 5 h (OD_{600} of about 0.5) at the final concentration of 0.1%. Samples were taken at times shown after the addition of arabinose, separated into medium and cell fractions and β-galactosidase activities in both fractions were measured as described in Materials and methods. The activity of the medium fraction is expressed as the percentage of total activity. Open columns and gray columns are W3110N *lon*::*kan* cells harboring pBAD24 and W3110N *lon*::*kan* cells harboring pBAD-*sulA*, respectively. Error bars are ±SD.

(a)

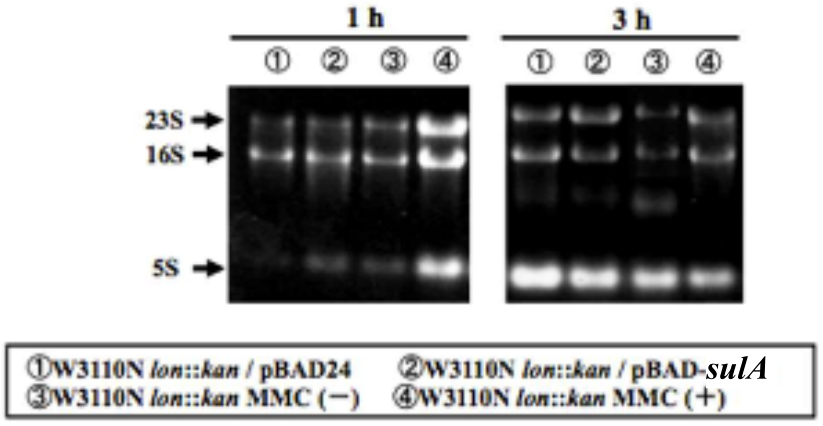

(b)

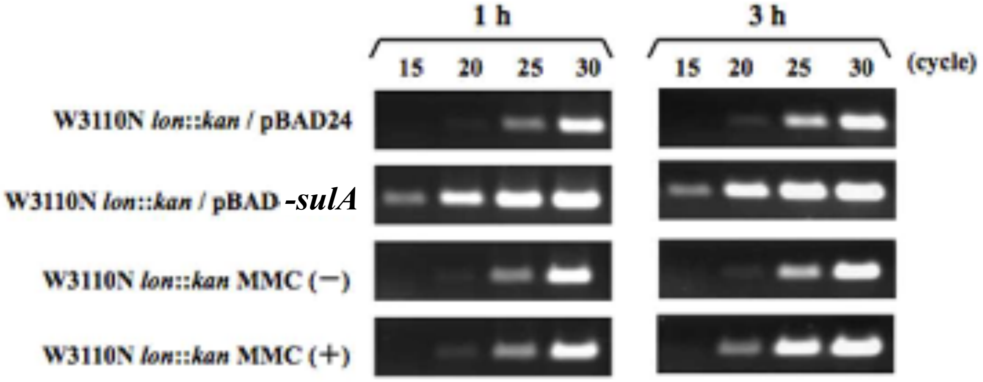

Figure S2 Expression level of *sulA* under the condition with MMC or with overexpression of *sulA*.
W3110N *lon*::*kan* cells harboring pBAD24 or pBAD-*sulA* were grown in LB medium containing ampicillin and kanamycin. W3110N *lon*::*kan* cells were grown in LB medium containing kanamycin. L-Arabinose and MMC were added at OD_{600} of about 0.5 at the final concentrations of 0.1% and 0.1 μg/ml, respectively. Total RNA was then isolated from cells at 1 h and 3 h after the induction and subjected to RT-PCR as described in Materials and methods. (a) Total RNA (10 μg) was run and separated by 1.2% agarose gel electrophoresis. (b) RT-PCR was performed with specific primers for *sulA*. After RT reaction, PCR reaction was performed, and the products obtained every 5 cycles from 15 to 30 cycles were analyzed by 0.9% agarose gel electrophoresis.

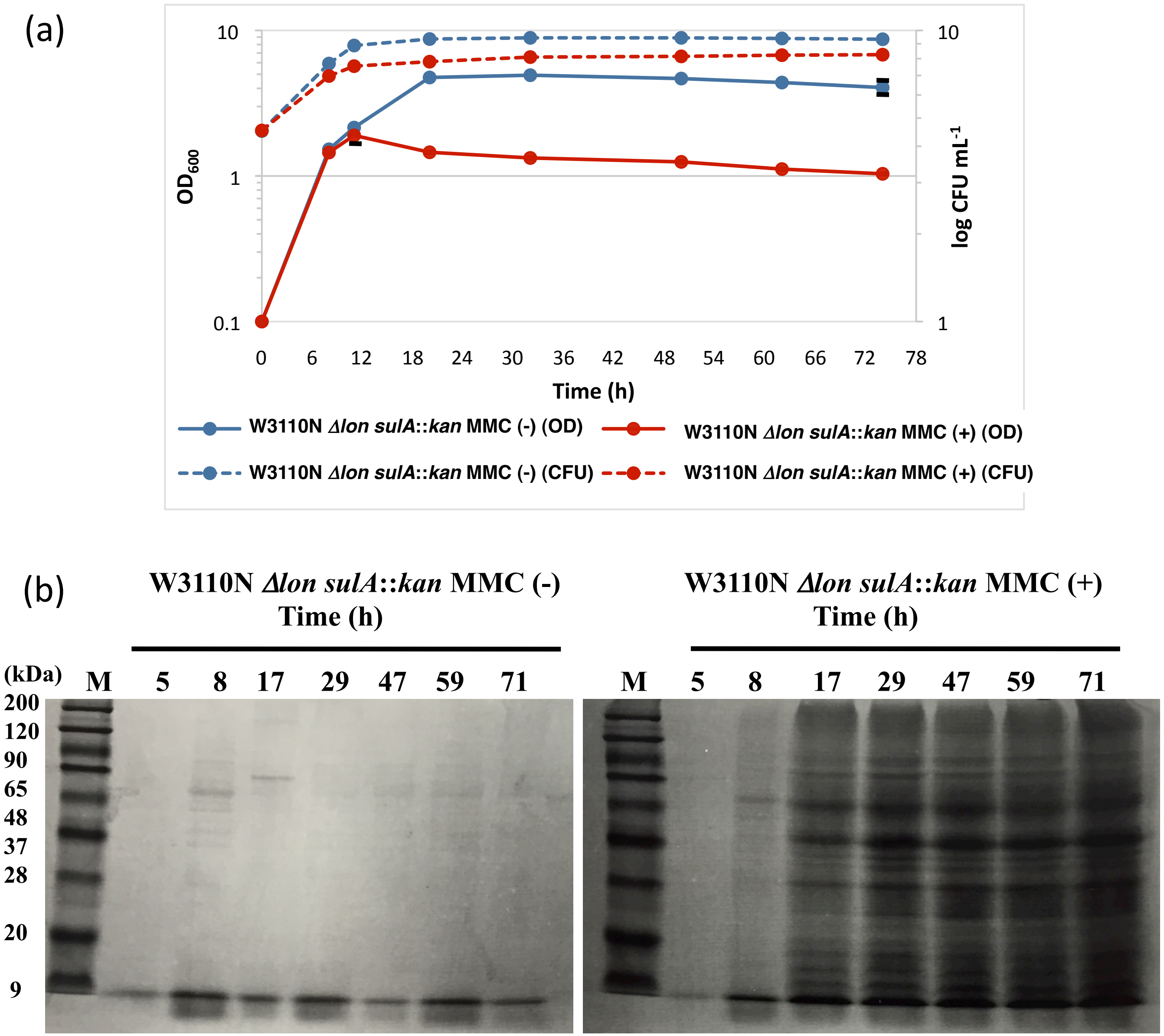

Figure S3. Effects of mitomycin C on cell growth and cell lysis. W3110N *Δlon sulA*::*kan* cells were grown in LB medium containing kanamycin, and mitomycin C (MMC) was added at 5 h (OD_{600} of about 0.5) at the final concentrations of 0.1 mg/ml. Cell turbidity (straight lines) and colony forming unit (CFU) (dotted lines) were determined. Red circles and blue circles in (a) represent conditions with and without MMC, respectively. Proteins from the medium fraction were recovered and subjected to SDS-PAGE as described in Materials and methods. Lane M is molecular markers.

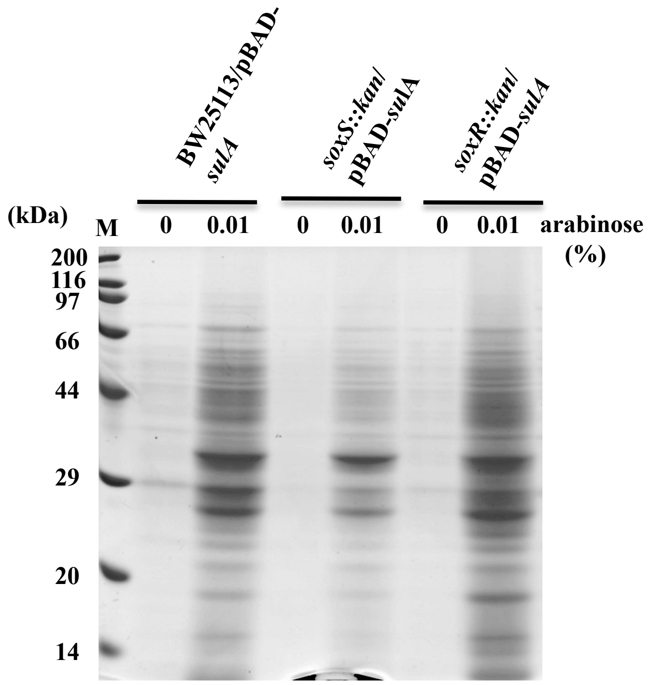

Figure S4 Effect of disrupted mutation of *soxR* on SDCL.
BW25113, BW25113 *soxS*::*kan*, and BW25113 *soxR*::*kan* cells harboring pBAD-*sulA* were grown in LB medium containing ampicillin. L-Arabinose was added at OD_{600} of about 0.5 at the final concentration of 0.1%. Portions of cultures were taken at 24 h after induction. Sample preparation for SDS-PAGE was performed as described in Materials and methods .

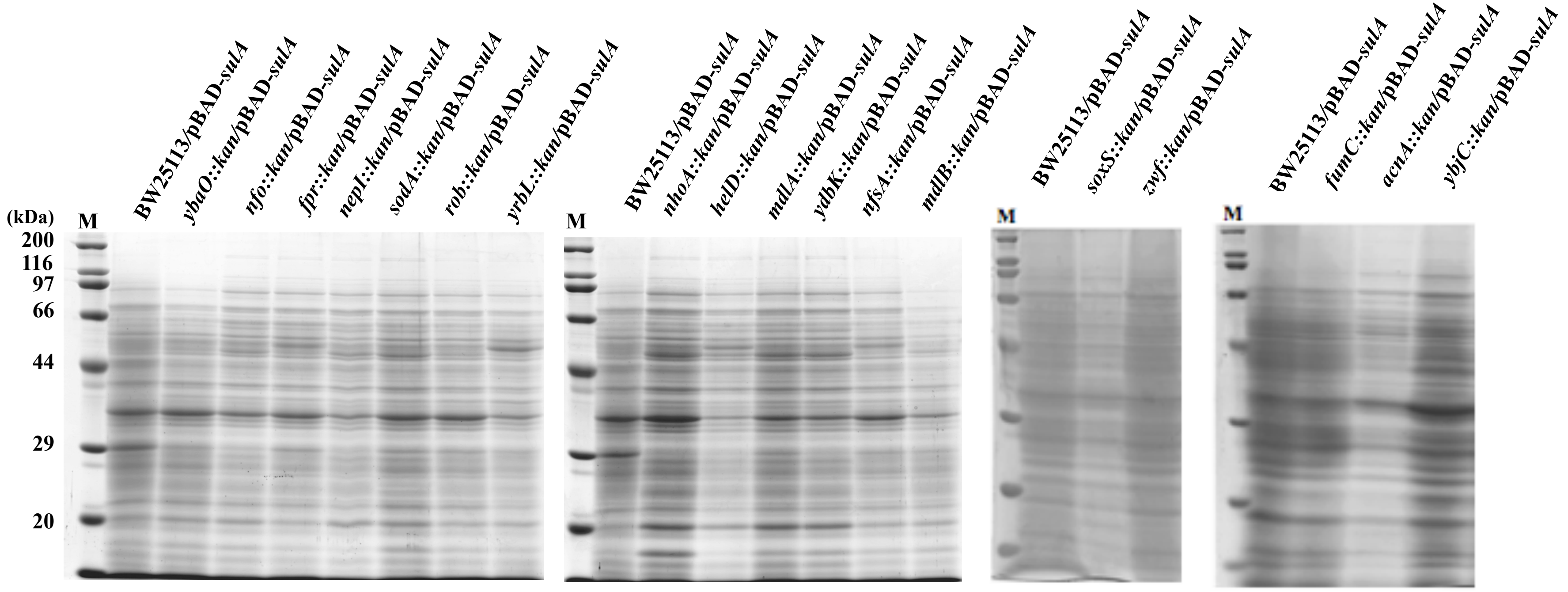

Figure S5 Effects of disrupted mutation of 16 SoxS regulon genes on SDCL.
Gene-disrupted derivatives of BW25113 harboring pBAD-*sulA* and BW25113 harboring pBAD-*sulA* were grown in LB medium, and L-arabinose was added at OD_{600} of about 0.5 at the final concentration of 0.01%. Portions of cultures were taken at 48 h after induction. Sample preparation for SDS-PAGE was performed as described in Materials and methods. BW25113 *kan*::*helD* and BW25113 *kan*::*soxS* were controls.

(a) (b)

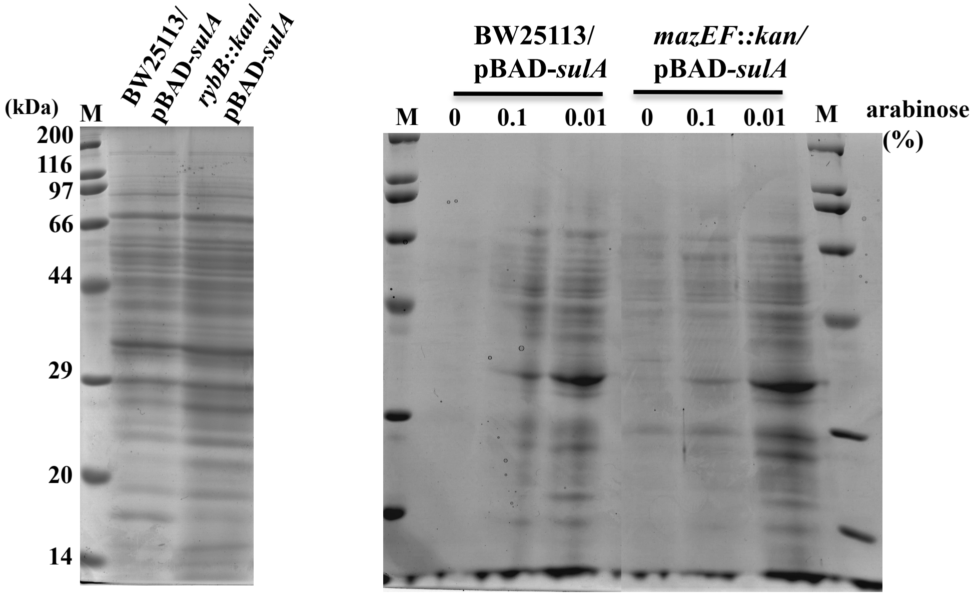

Figure S6 Effects of *ΔrybB* and *ΔmazEF* on SDCL.
(a) BW25113 and BW25113 *rybB*::*kan* cells harboring pCA24N-*sulA* were grown in LB medium containing ampicillin. IPTG was added at OD_{600} of about 0.5 at the final concentration of 0.1 mM. (b) BW25113 and BW25113 *mazEF*::*kan* cells harboring pBAD-*sulA* were grown in LB medium containing ampicillin. L-Arabinose was added at OD_{600} of about 0.5 at the final concentration of 0.1% or 0.01%. Portions of cultures were taken at 24 h after induction. Sample preparation for SDS-PAGE was performed as described in Materials and methods.

Table S1. Bacterial strains and plasmids

Strains / plasmids	Relevant property	References
Strains		
W3110N	IN(*rrnD–rrnE*) *rph-1rpoS*(33Q)	Kabir et al., 2004
BW25113	Δ*lacZ4787*:: *rrnB-3 hsdR514* DE(*araBAD*)*567* DE(*rhaBAD*)568 *rph-1*	Datsenko and Wanner (2000)
BW25113 *lon*::*kan*	BW25113 *Δlon*::*kan*	Baba *et al.* (2006)
BW25113 *soxS*::*kan*	BW25113 *ΔsoxS*::*kan*	Baba *et al.* (2006)
BW25113 *helD*::*kan*	BW25113 *ΔhelD*::*kan*	Baba *et al.* (2006)
BW25113 *rybB*::*kan*	BW25113 *ΔrybB*::*kan*	Baba *et al.* (2006)
BW25113 *sodA*::*kan*	BW25113 *ΔsodA*::*kan*	Baba *et al.* (2006)
BW25113 *acnA*::*kan*	BW25113 *ΔacnA*::*kan*	Baba *et al.* (2006)
BW25113 *nhoA*::*kan*	BW25113 *ΔnhoA*::*kan*	Baba *et al.* (2006)
BW25113 *mdlA*::*kan*	BW25113 *ΔmdlA*::*kan*	Baba *et al.* (2006)
BW25113 *mdlB*::*kan*	BW25113 *ΔmdlB*::*kan*	Baba *et al.* (2006)
BW25113 *fpr*::*kan*	BW25113 *Δfpr*::*kan*	Baba *et al.* (2006)
BW25113 *nfo*::*kan*	BW25113 *Δnfo*::*kan*	Baba *et al.* (2006)
BW25113 *ybjC*::*kan*	BW25113 *ΔybjC*::*kan*	Baba *et al.* (2006)
BW25113 *rob*::*kan*	BW25113 *Δrob*::*kan*	Baba *et al.* (2006)
BW25113 *nepI*::*kan*	BW25113 *ΔnepI*::*kan*	Baba *et al.* (2006)
BW25113 *fumC*::*kan*	BW25113 *ΔfumC*::*kan*	Baba *et al.* (2006)
BW25113 *zwf*::*kan*	BW25113 *Δzwf*::*kan*	Baba *et al.* (2006)
BW25113 *ybaO*::*kan*	BW25113 *ΔybaO*::*kan*	Baba *et al.* (2006)
BW25113 *acnA*::*kan*	BW25113 *ΔacnA*::*kan*	Baba *et al.* (2006)
BW25113 *yrbL*::*kan*	BW25113 *ΔyrbL*::*kan*	Baba *et al.* (2006)
BW25113 *nfsA*::*kan*	BW25113 *ΔnfsA*::*kan*	Baba *et al.* (2006)
BW25113 *mazEF*::*kan*	BW25113 *ΔmazEF*::*kan*	This study
W3110N *lon*::*kan*	W3110N *Δlon*::*kan*	This study
W3110N *Δlon*	W3110N *Δlon*	This study
W3110N *Δlon sulA*::*kan*	W3110N *Δlon ΔsulA*::*kan*	This study
Plasmids		
pBAD24	*araBAD* promoter, amp^r	Guzman *et al.*, (1995)

pBAD-*sulA*	pBAD24 bearing *sulA*	This study
pBAD-*soxS*	pBAD24 bearing *soxS*	This study
pBAD-*lpxC*	pBAD24 bearing *lpxC*	This study
pBAD-*helD*	pBAD24 bearing *helD*	This study
pCA24N	T-5 *lac* promoter, *lacI*^{q}, *cml*^{r}	Kitagawa *et al*. (2005)
pCA24N-*sulA*	pCA24N bearing *sulA*	This study
pCA24N-*soxS*	pCA24N bearing *soxS*	This study
pCA24N-*helD*	pCA24N bearing *helD*	Kitagawa *et al*. (2005)
pCA24N-*fldA*	pCA24N bearing *fldA*	Kitagawa *et al*. (2005)
pCA24N-*ribA*	pCA24N bearing *ribA*	Kitagawa *et al*. (2005)
pCA24N-*cspB*	pCA24N bearing *cspB*	Kitagawa *et al*. (2005)
pCA24N-*lpxC*	pCA24N bearing *lpxC*	Kitagawa *et al*. (2005)

Table 2S Primers used in this study

Primer	Sequence
For construction of pCA24N recombinants	
F-soxS	GCCTCCCATCAGAAAATTATTCAGGATCTTATCG
R-soxS	TTGCGGCCGCTTACAGGCGGTGGCGAT
F-sulA	GCCTACACTTCAGGCTATGCACATCG
R-sulA	TTGCGGCCGCTTAATGATACAAATTAGAGTGAATTTTTAGCC
For construction of pBAD24 recombinants	
F-sulA	TTGAATTCGGGCTGGATTGATTATG
R-sulA	TTCTGCAGTCACGACTGAAAGCATT
F-soxS	TTGAATTCACCATGTCCCATCAGAAAATTATTCAGG
R-soxS	TTAAGCTTTTACAGGCGGTGGCGATAATC
F-lpxC	TTGAATTCACCATGATCAAACAAAGGACACTTAAAC
R-lpxC	TTAAGCTTTTATGCCAGTACAGCTGAAGGC
F- helD	TGCTAGCAGGAGGAATTCACCATGGAACTGAAAGCGACAAC
R- helD	TTAAGCTTTTACGGTTTTCTCGCCACCGGCACATCCAG
For confirmation of W3110N *lon*::*kan* or W3110N *Δlon sulA*::*kan* by PCR	
lon-F	GTCCGACAAAGCAAGCG
lon-R	CATCAGTAGATGCGCCC
For construction of W25113 *mazEF*::*kan*	
ΔmazEF-F	TTGATATATACTGTATCTACATATGATAGCGGTTTGAGGAAAGG GTTATGGTGTAGGCTGGAGCTGCTTCG
ΔmazEF-R	GTGACCAGAATAGAAGTGAGTTAGTAACACTACCCAATCAGTA CGTTAATATTCCGGGGATCCGTCGACC
For confirmation of W25113 *mazEF*::*kan* by PCR	

ΔmazEF-U	GTTGTTACGTGATATCACGACCATT
ΔmazEF-D	TAAAACACCACCTGGAATAGCAGAT
For RT-PCR	
sulA-F	GCACATCGTTCTTCGTC
sulA-R	AACCCCATAGCGTTACC

Table S3. Significantly up-regulated genes by overexpression of *sulA*

Gene	b-number	Ratio[a]	Function of product
cspB	b1557	5.141	Cold shock protein CspB
cyoA	b0432	3.719	Cytochrome *o* ubiquinol oxidase subunit I
cyoB	b0431	3.299	Cytochrome *o* ubiquinol oxidase subunit I
cyoC	b0430	3.948	Cytochrome *o* ubiquinol oxidase subunit III
cyoD	b0429	3.669	Cytochrome o ubiquinol oxidase subunit IV
cyoE	b0428	2.495	Protoheme IX farnesyltransferase (haeme O biosynthesis)
fis	b3261	2.291	Global DNA-binding transcriptional dual regulator
flhC	b1891	2.122	DNA-binding transcriptional regulator with FlhD
fxsA	b4140	2.214	Inner membrane protein
gpp	b3779	2.287	Guanosine pentaphosphatase/exopolyphosphatase
helD	b0962	25.237	DNA Helicase IV (DNA repairing)
infA	b0884	2.552	Translation initiation factor IF-1
lpdA	b0116	2.182	Lipoamide dehydrogenase, E3 component is part of three enzyme complexes
mukB	b0924	2.390	Fused chromosome partitioning proteins
mutT	b0099	2.196	Nucleoside triphosphate pyrophosphohydrolase, marked preference for dGTP
narG	b1224	3.221	Nitrate reductase 1, alpha subunit
narH	b1225	2.718	Nitrate reductase 1, beta (Fe-S) subunit
narJ	b1226	2.282	Molybdenum-cofactor-assembly chaperone subunit (delta subunit) of nitrate reductase 1
ndk	b2518	2.178	Multifunctional nucleoside diphosphate kinase, apyrimidinic endonuclease, and 3'-phosphodiesterase
nuoF	b2284	2.412	NADH:ubiquinone oxidoreductase, chain F
nuoG	b2283	2.111	NADH-quinone oxidoreductase chain G
nusA	b3169	2.190	Transcription termination/antitermination L factor
pstS	b3728	2.181	Phosphate transporter subunit
rho	b3783	2.037	Transcription termination factor Rho
rplB	b3317	2.192	50S ribosomal protein L2
rplC	b3320	2.776	50S ribosomal protein L3

rplM	b3231	2.207	50S ribosomal protein L13
rplQ	b3294	2.190	50S ribosomal subunit protein L17
rplV	b3315	2.206	50S ribosomal subunit protein L22
rplW	b3318	2.357	50S ribosomal protein L23
rpmA	b3185	2.089	50S ribosomal protein L27
rpmC	b3312	2.155	50S ribosomal protein L29
rpmD	b3302	2.220	50S ribosomal subunit protein L30
rpmI	b1717	2.192	50S ribosomal subunit protein L35
rpsC	b3314	2.187	30S ribosomal subunit protein S3
rpsF	b4200	2.389	30S ribosomal subunit protein S6
rpsI	b3230	2.375	30S ribosomal subunit protein S9
rpsJ	b3321	2.154	30S ribosomal protein S10
rpsK	b3297	2.521	30S ribosomal protein S11
rpsO	b3165	2.215	30S ribosomal protein S15
rpsS	b3316	2.726	30S ribosomal protein S19
sdiA	b1916	2.144	DNA-binding transcriptional activator(transcriptional regulator of *ftsQAZ* gene cluster)
secY	b3300	2.502	SecYEG protein translocase auxillary subunit
sodA	b3908	2.308	Superoxide dismutase [Mn]
soxS	b4062	2.269	DNA-binding transcriptional dual regulator
speD	b0120	2.850	S-adenosylmethionine decarboxylase
speE	b0121	2.572	Spermidine synthase
spr	b2175	2.109	Predicted peptidase, outer membrane lipoprotein
sulA	b0958	152.0	SOS cell division inhibitor
thrC	b0004	3.916	Threonine synthase
tpx	b1324	2.305	Lipid hydroperoxide peroxidase
yaaJ	b0007	2.775	Putative transporter protein (sodium transport)
yafK	b0224	2.216	Hypothetical protein (DNA topological change)
ycjX	b1321	2.177	Conserved hypothetical protein with nucleoside triphosphate hydrolase domain
ydbA	b1405	2.538	Hypothetical protein YdbA fragment
yejG	b2181	2.470	Hypothetical protein

yejL	b2187	2.341	Hypothetical protein
yfhL	b2562	3.870	Predicted 4Fe-4S cluster-containing protein
yhcN	b3238	2.441	Hypothetical protein YhcN precursor
yifB	b3765	3.927	Predicted bifunctional protein, enzyme and transcriptional regulator
ykgK	b0294	2.094	Predicted regulator
ylaC	b0458	2.711	Predicted inner membrane protein
yliH	b0836	2.093	Hypothetical protein

[a] Expression ratios of more than 2 fold. Values are the average of ratios of the *sulA* overexpression sample to the control sample from at least two independent spots.

Table S4. Significantly down-regulated genes by overexpression of *sulA*

Gene	b-number	Ratio[a]	Function of product
aceA	b4015	0.374	Isocytorate lyase, glyoxylate cycle
appY	b0564	0.441	DNA-binding transcriptional activator
borD	b0557	0.397	predicted lipoprotein
elaB	b2266	0.403	Hypothetical protein (cell growth and/or maintenance)
fcl	b2052	0.261	Bifunctional GDP-fucose synthetase: GDP-4-dehydro-6-deoxy-D-mannose epimerase and GDP-4-dehydro-6-L-deoxygalactose reductase
feaR	b1384	0.455	DNA-binding transcriptional regulator
fecD	b4288	0.369	Iron-dicitrate transporter subunit
gadA	b3517	0.160	Glutamate decarboxylase A, PLP-dependent
gadB	b1493	0.160	Glutamate decarboxylase B, PLP-dependent
gadE	b3512	0.303	DNA-binding transcriptional activator
galU	b1236	0.332	Glucose-1-phosphate uridylyltransferase
glcG	b2977	0.381	Hypothetical protein
gmd	b2053	0.337	GDP-D-mannose dehydratase, NAD(P)-binding
intR	b1345	0.388	Integrase
kch	b1250	0.487	Voltage-gated potassium channel
malZ	b0403	0.441	Maltodextrin glucosidase
mhpF	b0351	0.453	Acetaldehyde-CoA dehydrogenase II, NAD-binding
msyB	b1051	0.441	Hypothetical protein
otsA	b1896	0.314	Trehalose-6-phosphate synthase
slp	b3506	0.298	Outer membrane lipoprotein
ugd	b2028	0.257	UDP-glucose 6-dehydrogenase
wcaA	b2059	0.356	Predicted glycosyl transferase
wcaB	b2058	0.406	Predicted acyl transferase
wcaF	b2054	0.217	Predicted acyl transferase
yaeR	b0187	0.357	Predicted lyase
yaiA	b0389	0.369	Hypothetical protein
ybgS	b0753	0.173	Hypothetical protein

ycaC	b0897	0.385	Predicted hydrolase
yceK	b1050	0.421	Predicted lipoprotein
ydeI	b1536	0.321	Hypothetical protein
ydhT	b1669	0.457	Hypothetical protein
yeaO	b1792	0.375	Hypothetical protein
yebQ	b1828	0.463	Predicted transporter
yecT	b1877	0.371	Hypothetical protein
yfaP	b2225	0.312	hypothetical protein
ygdI	b2809	0.331	Hypothetical lipoprotein
ygdR	b2833	0.459	Hypothetical protein
yghA	b3003	0.346	Predicted glutathionylspermidine synthase, with NAD(P)-binding Rossmann-fold domain
yhhH	b3483	0.354	Hypothetical lipoprotein
yiaB	b3563	0.260	conserved inner membrane protein
yigF	b3817	0.181	Conserved inner membrane protein
yigG	b3818	0.281	Predicted inner membrane protein
yjbD	b4023	0.461	Hypothetical protein
yjbE	b4026	0.178	Hypothetical protein
yjbF	b4027	0.194	Predicted lipoprotein
yjbJ	b4045	0.431	Predicted stress response protein
yjdJ	b4127	0.367	Predicted acyltransferase with acyl-CoA N-acyltransferase domain
ymfl	b1143	0.323	Predicted DNA-binding transcriptional regulator
yqeI	b2847	0.388	Predicted transcriptional regulator
yqeJ	b2848	0.328	Hypothetical protein
ytfE	b4209	0.165	Predicted regulator of cell morphogenesis and cell wall metabolism

[a]Expression ratios of less than 0.5 fold.Values are the average of ratios of the *sulA* overexpression sample to the control sample from at least two independent spots.

Table S5. Summary of significantly up-regulated and down-regulated genes by overexpression of *sulA*

	Classification	Up-regulated gene[a]	Down-regulated gene[b]
A	Amino acid metabolism	*thrC*	*wcaB*
B	Cell envelope	*flhC, spr*	*ygdI*
C	Cellular process	*mukB, secY, sodA, tpx*	*msyB, otsA*
D	Central intermediary metabolism	*speD, speE*	*aceA, appY, fcl, gadA, gadB, glcG, gmd, ugd*
E	Energy metabolism	*cyoA, cyoB, cyoC, cyoD, cyoE, lpdA, narG, narH, narJ, nuoF, nuoG*	*galU, malZ, mhpF, wcaF*
F	Nucleotide metabolism	*mutT, ndk*	
G	Regulatory functions	*gpp, sdiA, soxS*	*feaR, gadE*
H	Replication	*fis, helD*	
I	Transport/binding protein	*pstS*	*fecD, kch*
J	Translation	*infA, rplB, rplC, rplM, rplQ, rplV, rplW, rpmA, rpmC, rpmD, rpmI, rpsC, rpsF, rpsI, rpsJ, rpsK, rpsO, rpsS*	
K	Transcription	*nusA, rho*	
L	Other categories	*cspB, fxsA*	*borD, intR, wcaA*
M	Hypothetical	*yaaJ, yafK, ycjX, ydbA, yejG, yejL, yfhL, yhcN, yifB, ykgK, ylaC, yliH*	*elaB, slp, yaeR, yaiA, ybgS, ycaC, yceK, ydeI, ydhT, yeaO, yebQ, yecT, yfaP, ygdR, yghA, yhhH, yiaB, yigF, yigG, yjbD, yjbE, yjbF, yjbJ, yjdJ, ymfI, yqeI, yqwJ, ytfE*
	Total number of genes	62	51

[a] Up-regulated genes with expression ratios of more than 2 fold.

[b] Down-regulated genes with expression ratios of less than 0.5 fold.
